# Dysfunctional labor: Case definition & guidelines for data collection, analysis, and presentation of immunization safety data

**DOI:** 10.1016/j.vaccine.2017.01.050

**Published:** 2017-12-04

**Authors:** Adeline A. Boatin, Linda O. Eckert, Michel Boulvain, Chad Grotegut, Barbra M. Fisher, Jay King, Marie Berg, Richard M.K. Adanu, Uma Reddy, Jason J.S. Waugh, Manish Gupta, Sonali Kochhar, Sara Kenyon, The Brighton Collaboration Dysfunctional Labor Working Group

**Affiliations:** a Department of OB/GYN, Massachusetts General Hospital, Harvard Medical School, Boston, USA; b University of Washington, Seattle, WA, USA; c Service d'obstétrique, Maternité HUG, Switzerland; d Division of Maternal-Fetal Medicine, Duke University, USA; e Maternal Fetal Medicine, Northwest Perinatal Centre, Women's Healthcare Associates, LLC, Portland, USA; f Sanofi Pasteur, USA; g Institute of Health Care Science, University of Gothenburg, Gothenburg, Sweden; h University of Ghana School of Public Health, Ghana; i Pregnancy and Perinatology Branch, Eunice Kennedy Shriver National Institute of Child Health and Human Development, National Institutes of Health, USA; j Royal Victoria Infirmary, Newcastle Upon Tyne, United Kingdom; k Barts Health NHS Trust, London, United Kingdom; l Global Healthcare Consulting, India; m Reader in Evidence Based Maternity Care, Institute for Applied Health Research, University of Birmingham, United Kingdom; n Erasmus University Medical Center, Rotterdam, The Netherlands

**Keywords:** Dysfunctional labor, Prolonged labor, Abnormal labor, Immunization, Guidelines, Case definition

## 1 Preamble

### 1.1 Need for developing case definitions and guidelines for data collection, analysis, and presentation for dysfunctional labor as an adverse event following immunization

Vaccination during pregnancy is recommended for both maternal and neonatal benefit against a number of potential infections. The tetanus toxoid, reduced diphtheria toxoid and acellular pertussis vaccine is now routine recommended for pregnant women in each pregnancy not only for maternal benefit, but to confer passive antibody transfer to the newborn until infant immunizations can be given [1]. Influenza vaccinations are also strongly recommended for any pregnant woman, or women who might become pregnant during influenza seasons [2]. The safety of both these vaccinations has been well established. Efforts to develop new vaccinations for use during pregnancy represent a new opportunity to prevent common maternal and neonatal infections with severe morbidity and mortality. There is growing interest and research around maternal immunization against both Group B streptococcus (GBS) and Respiratory syncytial virus (RSV) as a public health strategy to prevent neonatal and infant infections worldwide [3], [4].

Establishing the safety profile of any new vaccination requires careful surveillance of potential adverse effects and consistent terminology and definitions across context and time. The World Health Organization (WHO) defines an ‘adverse event following immunization’ (AEFI) as “any untoward medical occurrence which follows immunization and, which does not necessarily have a causal relationship with the use of the vaccine. The adverse event may be an unfavorable or unintended sign, an abnormal laboratory finding, a symptom or disease” [5]. Recognizing that vaccination is often temporally related to many events, abnormal findings or diseases, causality assessment between an AEFI and vaccination requires further rigorous assessment and study. Monitoring of a broad array of events, including those without established or suspected links to vaccine can therefore provide the initial basis for data with which such causality can be proven or disproven.

Dysfunctional labor is relatively common occurrence during the intrapartum stage of pregnancy. Incidence estimates vary due to differences in definitions, but approximately 20% of labors are thought to be affected by this condition [6]. Though there are no reported links between dysfunctional labor and immunization, the measurement of this potential complication in association with vaccination is important to establish vaccine safety. Despite being relatively common, there is a lack of consensus on the criteria for the diagnosis of dysfunctional labor. Guidelines from professional obstetric societies differ in both the criteria used to define this process and when intervention should occur (Table 1). The Brighton Collaboration has been developing standardized definitions for use in vaccine trials since 2001 [7]. To further consistent terminology and definitions of outcomes and adverse events typically reported in vaccine trials, specifically for maternal immunization, standardized definitions of common obstetric outcomes are needed. The goal of this working group was therefore to provide a case definition for this term to facilitate surveillance and case ascertainment in vaccine trials.

**Table 1:** Summary of professional guidelines.

Professional Organization	Year Published	First Stage		Second Stage	
		Nulliparous	Multiparous	Nulliparous	Multiparous
NICE [29]	2014	Normal: 8–18 h	Normal: 5–12 h	Birth expected within 3 h of start of active second stage	Birth expected within 2 h of start of active stage
		Suspected delay: <2 cm in 4 h, with delay confirmed with progress of less than 1 cm h later	Delay: <2 cm in 4 h OR slowing in progress of labor	Delay: 2 or more hours	Delay: 1 or more hours
ACOG/SMFM [11]	2014	Normal < 20 h	Normal < 14 h	No maximum time frame	No maximum time frame
		Arrest: 6 cm dilation and 4 h or more of adequate contractions or 6 h or more of inadequate contractions	Arrest: 6 cm dilation and 4 h or more of adequate contractions or 6 h or more of inadequate contractions	Permit at least	Permit at least
				3 h of pushing	2 h of pushing
RANZCOG [30]	2014	Prolonged if: <1 cm/h in active phase	Prolonged if: <1 cm/h in active phase	>2 h	>1 h
WHO [31]	2014	<0.5 cm to 1 cm/h during the active phase	<0.5 cm to 1 cm/h during the active phase	N/A	N/A
SOGC [32]	1995	<0.5 cm/h over a 4 h period	<0.5 cm/h over a 4 h period	2 h if no regional anesthesia	N/A
FIGO [33]	2012	N/A	N/A	No more than 3 h of active pushing	No more than 2 h of active pushing

Labor is typically divided into three stages. The first stage of labor marks the onset of labor until full dilation of the cervix; the second stage, full dilation until delivery of the fetus, and the third, delivery of the placenta. In the 1950s, Friedman first described the first stage dividing this into latent and active phases of labor [8], [9]. His work first demonstrated the broad range of labor duration experienced by women and until recently provided the basis for defining normal progress and length of labor limits of normal labor duration. Recent evidence, however, from a larger more diverse population of women have challenged these historical durations [10].

Dysfunctional or prolonged labor refers to prolongation in the duration of labor, typically in the first stage of labor. Diagnosis of delay in labor is dependent on careful monitoring of uterine contraction intensity, duration and frequency, cervical dilation and descent of the fetus through the pelvis. Dysfunctional labor can be an important contributor to maternal and perinatal mortality and morbidity if it remains unrecognized and untreated when needed. On the other hand, pre-emptive diagnosis of dysfunctional labor may lead to unnecessary interventions. Labor dysfunction is a leading indication for primary caesarean section and there is concern, that an over diagnosis may be a contributor to high and rising caesarean section rates [11].

The pathophysiology of dysfunctional labor is multifactorial and complex and yet to be fully elucidated. Often, the exact etiology of dysfunctional labor is unknown. Broadly, etiology can be categorized into uterine contractile dysfunctions and abnormalities in the cephalopelvic ratio (i.e. the relation of the fetal size, presentation and position to the maternal pelvis). Both these causes can be influenced by a number of genetic and environmental factors including but not limited to maternal and gestational age, pre-pregnancy body mass index, pregnancy weight gain, physical activity, medical co-morbidities, parity, and obstetric complications (pre-eclampsia, premature rupture of membranes, chorio-amnionitis, placental abruption) [12], [13], [14], [15].

### 1.2 Methods for the development of the case definition and guidelines for data collection, analysis, and presentation for dysfunctional labor as an adverse events following immunization

Following the process described in the overview paper [16] as well as on the Brighton Collaboration Website http://www.brightoncollaboration.org/internet/en/index/process.html, the Brighton Collaboration *Dysfunctional Labor Working Group* was formed in 2015 and included members of clinical, academic, public health and industry background. The composition of the working and reference group as well as results of the web-based survey completed by the reference group with subsequent discussions in the working group can be viewed at: http://www.brightoncollaboration.org/internet/en/index/working_groups.html.

To guide the decision-making for the case definition and guidelines, a literature search for publications in any language was performed using Medline, Embase and the Cochrane Libraries, including the terms dysfunctional, prolonged, delayed, obstructed, abnormal, augmented labor, arrest of dilation, labor dystocia AND ‘vaccination’ or ‘vaccine’ or ‘immunization’ OR ‘immunize’ OR ‘inoculation’*.* The search resulted in the identification of 172 references. All abstracts were screened for possible reports of dysfunctional labor following immunization. Two full text articles with potentially relevant material were reviewed in more detail, in order to identify studies using case definitions or, in their absence, providing clinical descriptions of the case material [17], [18]. This review resulted in no articles providing case reports or case definitions of dysfunctional labor following immunization.

To further guide decision-making process, guidelines from major professional obstetric societies were reviewed and definitions of dysfunctional or prolonged labor summarized and provided to members of the working group for review (Table 1).

### 1.3 Rationale for selected decisions about the case definition of dysfunctional labor as an adverse event following immunization

Our focus throughout this process is to provide criteria for diagnostic certainty for the purpose of case definition rather than for the identification of time frames for intervention or management changes. This is a marked difference from the definitions provided in most guidelines where a timeframe or criteria are used to suggest when intervention and management changes should occur. Thus, a case meeting the definitions provided may or may not warrant intervention, however it is not within the purview of this working group to provide such recommendations.

We recommend criteria used in case ascertainment as defined below are restricted to term singleton pregnancies, i.e. at or after 37 completed weeks and before 42 weeks of pregnancy. The pathophysiology and course of labor in preterm, previable pregnancies or postdate pregnancies was felt to be sufficiently different that separate terminology and case definitions should apply. Both preterm labor and pregnancy loss have been separately defined by working groups though the Brighton collaboration [19], [20]. Despite similarities between labor in multiple vs. singleton gestations the working group felt the interaction between vaccination in a these different gestations may be different and thus warrant a separate consideration.

Definitions were formed separately for both the first stage and second stage of labor. Within the first stage we choose to focus on a definition of dysfunction once the established or active labor is reached. The definitions, therefore do not address potential dysfunction or protraction of the latent phase in the first stage of labor. Established labor describes the onset of active labor, (regular contractions and a cervical dilation of 4 centimeter (cm)). This is distinct from the active phase of the first stage as described by Zhang et al. where there is acceleration in the rate of dilation at 6 cm. This stage of cervical dilation has since been used by United States professional societies as the basis for recommendations on a point before which unnecessary intervention should be avoided, rather than as a definition of established labor [21], [10]. Established labor was also chosen as the starting time point as it includes all labors once they are established, regardless of whether they were initially induced or spontaneous. The requirement for regular contractions and cervical dilation of at least 4 cm would mean that an induced labor that begins with foley catheter cervical ripening and achieves a mechanical dilation of 4 cm, but without regular contractions is not considered in established labor. Similarly a multiparous woman with a cervix dilated to 4 cm but without any contractions would not be considered in established labor.

We chose not to include the use of regional analgesia as a separate category within our definitions for both first and second stages of labor. We recognize that during routine clinical practice, the diagnosis of dysfunctional labor and potential subsequent intervention is often adjusted in the presence of regional analgesia. Current evidence suggests no impact of regional anesthesia on the first stage [22]. We recognize literature that shows that the second stage is longer in women with regional anesthesia [22], however for the purposes of case definition the working group considered regional analgesia a risk factor for a prolonged second stage rather than warranting separate categorization. As noted above, the scope of these definitions are for case ascertainment alone and not to prescribe or prevent intervention. Thus by excluding regional anesthesia as an influence on the case definition we do not mean to suggest any change in management decisions that might occur in women who do undergo regional anesthesia.

In formulating a case definition that reflects diagnostic certainty we weighed specificity versus sensitivity. After reaching consensus, only two levels of definition were formed, both of which relate to diagnostic certainty around case definition, rather than clinical severity of a case. To make the diagnosis of dysfunctional labor, an examiner capable of reliably assessing uterine contractions, amniotic membrane status (intact vs. ruptured), cervical dilation, fetal station and a measure of time is required. The case definition has been formulated such that the level one definition is highly specific for the condition. As maximum specificity normally implies a loss of sensitivity, one additional diagnostic level has been included in the definition, offering a stepwise increase of sensitivity from level one to level two, while retaining an acceptable level of specificity at all levels. In this way it is hoped that all possible cases of dysfunctional labor can be captured. In the first stage of labor, certainty around ruptured membranes distinguishes between a level one and level two level of certainty. This second level of diagnostic certainty recognizes circumstances where the timing or certainty of rupture of membranes is unknown to the woman or her provider or is not documented in the medical record. In the second stage of labor, diagnostic certainty in level one and two are distinguished by certainty around the onset of active maternal effort i.e. pushing or visible baby after full dilation (cervix is reported at 10 cm or no longer felt around the presenting part). This allows for variations in practice where women are allowed passive descent of the fetal head after full dilation or in cases where the onset of active pushing after full dilation is not recorded. In both stages of labor, the working group determined a third level of definition would be not be specific enough to reliably measure cases of dysfunctional labor, therefore a third level of diagnostic certainty was not included.

#### 1.3.1 Influence of treatment on fulfillment of case definition

The Working Group decided against using “treatment” or “treatment response” towards fulfillment of dysfunctional labor case definition except in the second stage of labor. No distinction is made between spontaneous, augmented or induced labors, though recognizing, that induction may represent a risk factor for labor dysfunction. Similarly in the second stage of labor, no distinction is made for labors in which women receive regional anesthesia, though as noted above, it is recognized that this might represent a risk factor for prolonged second stage. We designed both level one and two definitions to be broad enough to include cases presenting differently due to appropriate and early treatment initiation.

An exception is made for intervention for delivery in the second stage of labor, either by operative vaginal delivery or caesarean delivery for the indication of failure to progress or arrest of descent. This exception was made as it was felt practice patterns exist where early intervention is performed in the second stage and exclusion of these cases could result in underreporting of dysfunctional labor.

#### 1.3.2 Timing post immunization

Specific time frames for the onset of symptoms following immunization are not included in this definition. Due to the lack of a reported link between dysfunctional labor and immunization, and no postulated biological plausibility for a link, we felt a restrictive time interval from immunization to onset of dysfunctional labor should not be an integral part of such a definition. Furthermore, labor often occurs outside the controlled setting of a clinical trial or hospital. In some settings it may be impossible to obtain a clear timeline of the event, particularly in less developed or rural settings. In order to avoid selecting against such cases, the Brighton Collaboration case definition avoids setting arbitrary time frames. Therefore, we recommend that details of this interval should be assessed and reported as described in the data collection guidelines.

### 1.4 Guidelines for data collection, analysis and presentation

As mentioned in the overview paper, the case definition is accompanied by guidelines which are structured according to the steps of conducting a clinical trial, i.e. data collection, analysis and presentation. Neither case definition nor guidelines are intended to guide or establish criteria for management of ill infants, children, or adults. Both were developed to improve data comparability.

### 1.5 Periodic review

Similar to all Brighton Collaboration case definitions and guidelines, review of the definition with its guidelines is planned on a regular basis (i.e. every three to five years) or more often if needed.

## 2 Case definition of dysfunctional labor

### 2.1 First stage of labor

For both levels of diagnostic certainty, the woman is in established labor defined by regular contractions and cervical dilation of at least 4 cm.

*Level 1 of diagnostic certainty*

Progress of less than 0.5 cm cervical dilation per hour, for at least 4 h,^3^ in women in established labor (i.e. have regular contractions and cervical dilation of at least 4 cm) and with confirmed ruptured membranes.^4^

*Level 2 of diagnostic certainty*

Progress of less than 0.5 cm cervical dilation per hour in women, for at least 4 h, with established labor (i.e. that is, regular contractions and cervical dilation of at least 4 cm) without certainty of ruptured membranes.

### 2.2 Second stage of labor

*Level 1 of diagnostic certainty*

*Nulliparous women:*

Full dilation^5^ of the cervix
AND
onset of the active stage (active maternal effort (i.e. pushing) OR visible baby)
AND
greater than 2 h of pushing
  OR use of instrument delivery for the indication of dystocia^6^^,^^7^
  OR caesarean delivery for the indication of dystocia (see footnote 7)

*Multiparous women:*

Full dilation of the cervix
AND
onset of the active stage (active maternal effort (i.e. pushing) OR visible baby)
AND
greater than 1 h of pushing
  OR use of instrument delivery for the indication of dystocia (see footnotes 6, 7)
  OR caesarean delivery for the indication of dystocia (see footnote 7)

*Level 2 of diagnostic certainty*

*Nulliparous women*

Full dilation of the cervix in any phase of the second stage
AND
no delivery within 3 h of full dilation
  OR use of instrument delivery for the indication of dystocia (see footnotes 6, 6)
  OR caesarean delivery for the indication of dystocia (see footnote 6)

*Multiparous women*

Full dilation of the cervix in any phase of the second stage
AND
no delivery within 3 h of full dilation
  OR use of instrument delivery for the indication of dystocia (see footnotes 6, 7)
  OR caesarean delivery for the indication of dystocia (see footnote 6)

## 3 Guidelines for data collection, analysis and presentation of dysfunctional labor

It was the consensus of the Brighton Collaboration *Dysfunctional Labor Working Group* to recommend the following guidelines to enable meaningful and standardized collection, analysis, and presentation of information about dysfunctional labor. However, implementation of all guidelines might not be possible in all settings. The availability of information may vary depending upon resources, geographical region, and whether the source of information is a prospective clinical trial, a post-marketing surveillance or epidemiological study, or an individual report of dysfunctional labor. Also, as explained in more detail in the overview paper in this volume, these guidelines have been developed by this working group for guidance only, and are not to be considered a mandatory requirement for data collection, analysis, or presentation.

### 3.1 Data collection

These guidelines represent a desirable standard for the collection of data on availability following immunization to allow for comparability of data, and are recommended as an addition to data collected for the specific study question and setting. The guidelines are not intended to guide the primary reporting of dysfunctional labor to a surveillance system or study monitor. Investigators developing a data collection tool based on these data collection guidelines also need to refer to the criteria in the case definition, which are not repeated in these guidelines.

Guidelines numbers below have been developed to address data elements for the collection of adverse event information as specified in general drug safety guidelines by the International Conference on Harmonization of Technical Requirements for Registration of Pharmaceuticals for Human Use [23] and the form for reporting of drug adverse events by the Council for International Organizations of Medical Sciences [24] and formulated by Jones et al. [25]. These data elements include an identifiable reporter and patient, one or more prior immunizations, and a detailed description of the adverse event, in this case, of dysfunctional labor following immunization. The additional guidelines have been developed as guidance for the collection of additional information to allow for a more comprehensive understanding of dysfunctional labor following immunization.

#### 3.1.1 Source of information/reporter

For all cases and/or all study participants, as appropriate, the following information should be recorded:

(1) Date of report.
(2) Name and contact information of person reporting^8^ and/or diagnosing the dysfunctional labor as specified by country-specific data protection law.
(3) Name and contact information of the investigator responsible for the subject, as applicable.
(4) Relation to the patient (e.g. Immunizer [clinician, nurse], family member [indicate relationship], other).

#### 3.1.2 Vaccine/Control

##### 3.1.2.1 Demographics

For all cases and/or all study participants, as appropriate, the following information should be recorded:

(5) Case/study participant identifiers (e.g. first name initial followed by last name initial) or code (or in accordance with country-specific data protection laws).
(6) Date of birth, age, and sex.
(7) For infants born to study participants: Gestational age and birth weight.

##### 3.1.2.2 Clinical and immunization history

For all cases and/or all study participants, as appropriate, the following information should be recorded:

(8) Past medical history, including hospitalizations, underlying diseases/disorders, pre-immunization signs and symptoms including identification of indicators for, or the absence of, a history of allergy to vaccines, vaccine components or medications; food allergy; allergic rhinitis; eczema; asthma.
(9) Any medication history (other than treatment for the event described) prior to, during, and after immunization including prescription and non-prescription medication as well as medication or treatment with long half-life or long-term effect (e.g. immunoglobulins, blood transfusion and immunosuppressants).
(10) Immunization history (i.e. previous immunizations and any adverse event following immunization (AEFI^9^)), in particular occurrence of an adverse event after a previous immunization.

#### 3.1.3 Details of the immunization

For all cases and/or all study participants, as appropriate, the following information should be recorded:

(11) Date and time of immunization(s).
(12) Description of vaccine(s) (name of vaccine, manufacturer, lot number, dose (e.g. 0.25 mL, 0.5 mL, etc.) and number of dose if part of a series of immunizations against the same disease).
(13) The anatomical sites (including left or right side) of all immunizations (e.g. vaccine A in proximal left lateral thigh, vaccine B in left deltoid).
(14) Route and method of administration (e.g. intramuscular, intradermal, subcutaneous, and needle-free (including type and size), other injection devices).
(15) Needle length and gauge.

#### 3.1.4 The adverse event

(16) For all cases at any level of diagnostic certainty and for reported events with insufficient evidence, the criteria fulfilled to meet the case definition should be recorded.

Specifically document:

(17) Clinical description of signs and symptoms of dysfunctional labor and if there was medical confirmation of the event (i.e. patient seen by physician).
(18) Date/time of onset of established labor,^10^ date/time of diagnosis of dysfunctional labor and final outcome including mode of delivery.^11^
(19) Time interval since immunization.
(20) Concurrent signs, symptoms, and diseases.
(21) Measurement/testing:
  • Values and units of routinely measured parameters (e.g. temperature, blood pressure) – in particular those indicating the severity of the event.
  • Method of measurement (e.g. type of thermometer, oral or other route, duration of measurement, etc.).
  • Results of laboratory examinations, surgical and/or pathological findings and diagnoses if present.
  • Include documentation of time and findings for cervical exam, onset of established labor, time of initiation and duration of regional analgesia if any, full dilation, onset of maternal pushing in the second stage, and time of delivery.
(22) Treatment or intervention given for dysfunctional labor, especially if medication dosing, if procedure – type of procedure.
(23) Outcome(see footnote 11) at last observation.
(24) Exposures other than the immunization 24 h before and after immunization (e.g. food, environmental) considered potentially relevant to the reported event.

#### 3.1.5 Miscellaneous/general

(25) The duration of surveillance for dysfunctional labor should be predefined based on
  • Biologic characteristics of the vaccine e.g. live attenuated versus inactivated component vaccines.
  • Biologic characteristics of the vaccine-targeted disease.
  • Biologic characteristics of dysfunctional labor including patterns identified in previous trials (e.g. early-phase trials).
  • Biologic characteristics of the vaccinee (e.g. nutrition, underlying disease like immunodepressing illness).
(26) The duration of follow-up reported during the surveillance period should be predefined likewise. It should aim to continue to resolution of the event, which in this case ascertainment would be after delivery is completed.
(27) Methods of data collection should be consistent within and between study groups, if applicable.
(28) Follow-up of cases should attempt to verify and complete the information collected as outlined in data collection guidelines 1–24.
(29) Investigators of patients with dysfunctional labor should provide guidance to reporters to optimize the quality and completeness of information provided.
(30) Reports of dysfunctional labor should be collected throughout the study period regardless of the time elapsed between immunization and the adverse event. If this is not feasible due to the study design, the study periods during which safety data are being collected should be clearly defined.

### 3.2 Data analysis

The following guidelines represent a desirable standard for analysis of data on dysfunctional labor to allow for comparability of data, and are recommended as an addition to data analyzed for the specific study question and setting.

(31) Reported events should be classified in one of the following five categories including the three levels of diagnostic certainty. Events that meet the case definition should be classified according to the levels of diagnostic certainty as specified in the case definition. Events that do not meet the case definition should be classified in the additional categories for analysis.

**Event classification in 5 categories**^12^

**Event meets case definition**

(1) Level 1: *Criteria as specified in the* on dysfunctional labor *case definition*
(2) Level 2: *Criteria as specified in the* on dysfunctional labor *case definition*

**Event does not meet case definition**

***Additional categories for analysis***

(3) Reported dysfunctional labor with insufficient evidence to meet the case definition.^13^
(4) Not a case of dysfunctional labor.^14^
(32) The interval between immunization and reported dysfunctional labor could be defined as the date/time of immunization to the date/time of onset (see footnote 10) of the first symptoms and/or signs consistent with the definition. If few cases are reported, the concrete time course could be analyzed for each; for a large number of cases, data can be analyzed in the following increments:

**Subjects with dysfunctional labor by Interval to Presentation**

**Table:** 

**Interval∗**	**Number**
<24 h after immunization	
24 h to <72 h after immunization	
72 h to <7 days after immunization	
7 days to <30 days after immunization	
More than 30 days	

**TOTAL**

(33) The duration of a possible dysfunctional labor could be analyzed as the interval between the date/time of the first signs consistent with the definition and the delivery of the fetus/es^15^ (see footnote 11). Whatever start and ending are used, they should be used consistently within and across study groups.
(34) The distribution of data (as numerator and denominator data) could be analyzed in predefined increments (e.g. measured values, times), where applicable. Increments specified above should be used. When only a small number of cases are presented, the respective values or time course can be presented individually.
(35) Data on dysfunctional labor obtained from subjects receiving a vaccine should be compared with those obtained from an appropriately selected and documented control group(s) to assess background rates of hypersensitivity in non-exposed populations, and should be analyzed by study arm and dose where possible, e.g. in prospective clinical trials. Sample size to evaluate exposed versus non-exposed populations should be calculated using background rates of dysfunctional labor in the population to be studied.

### 3.3 Data presentation

These guidelines represent a desirable standard for the presentation and publication of data on dysfunctional labor following immunization to allow for comparability of data, and are recommended as an addition to data presented for the specific study question and setting. Additionally, it is recommended to refer to existing general guidelines for the presentation and publication of randomized controlled trials, systematic reviews, and meta-analyses of observational studies in epidemiology (e.g. statements of Consolidated Standards of Reporting Trials (CONSORT), of Improving the quality of reports of meta-analyses of randomized controlled trials (QUORUM), and of Meta-analysis Of Observational Studies in Epidemiology (MOOSE), respectively) [26], [27], [28].

(36) All reported events of on dysfunctional labor should be presented according to the categories listed in guideline 32.
(37) Data on possible on dysfunctional labor events should be presented in accordance with data collection guidelines 1–24 and data analysis guidelines 31–35.
(38) Terms to describe on dysfunctional labor such as “low-grade”, “mild”, “moderate”, “high”, “severe” or “significant” are highly subjective, prone to wide interpretation, and should be avoided.
(39) Data should be presented with numerator and denominator (n/N) (and not only in percentages), if available.
Although immunization safety surveillance systems denominator data are usually not readily available, attempts should be made to identify approximate denominators. The source of the denominator data should be reported and calculations of estimates be described (e.g. manufacturer data like total doses distributed, reporting through Ministry of Health, coverage/population based data, etc.).
(40) The incidence of cases in the study population should be presented and clearly identified as such in the text.
(41) If the distribution of data is skewed, median and range are usually the more appropriate statistical descriptors than a mean. However, the mean and standard deviation should also be provided.
(42) Any publication of data on dysfunctional labor should include a detailed description of the methods used for data collection and analysis as possible. It is essential to specify:
  • The study design.
  • The method, frequency and duration of monitoring for on dysfunctional labor.
  • The trial profile, indicating participant flow during a study including drop-outs and withdrawals to indicate the size and nature of the respective groups under investigation.
  • The type of surveillance (e.g. passive or active surveillance).
  • The characteristics of the surveillance system (e.g. population served, mode of report solicitation).
  • The search strategy in surveillance databases.
  • Comparison group(s), if used for analysis.
  • The instrument of data collection (e.g. standardized questionnaire, diary card, report form).
  • Whether the day of immunization was considered “day one” or “day zero” in the analysis.
  • Whether the date of onset (see footnote 10) and/or the date of first observation^16^ and/or the date of diagnosis^17^ was used for analysis.
  • Use of this case definition for on dysfunctional labor, in the abstract or methods section of a publication.^18^

## Disclaimer

The findings, opinions and assertions contained in this consensus document are those of the individual scientific professional members of the working group. They do not necessarily represent the official positions of each participant’s organization (e.g. government, university, or corporation). Specifically, the findings and conclusions in this paper are those of the authors and do not necessarily represent the views of their respective institutions.

## Footnotes

3 “For at least 4 h” was added to ensure adequate time allowed for progression. Thus a case where progression is less than 0.5 cm in the first hour, but then subsequently speeds up would not meet the criteria for delayed or dysfunctional labor. It must be less than 0.5 cm/h averaged over 4 h.

4 Confirmed rupture of membranes is added to a level 1 of diagnostic certainty to exclude those women with advanced cervical exams who may have intermittent contractions but may not be in established labor. For example, the multiparous woman with an advanced cervical exam.

5 Full dilation of the cervix is described as 10 cm dilated, or no palpable cervix around the presenting part of the fetus.

6 Instrument delivery refers to delivery by forceps or vacuum/ventouse.

7 Dystocia indications include arrest of descent and failure to progress as opposed to indications for fetal well-being.

8 If the reporting centre is different from the vaccinating centre, appropriate and timely communication of the adverse event should occur.

9 An AEFI is defined as serious by international standards if it meets one or more of the following criteria: (1) it results in death, (2) is life-threatening, (3) it requires inpatient hospitalization or results in prolongation of existing hospitalization, (4) results in persistent or significant disability/incapacity, (5) is a congenital anomaly/birth defect, (6) is a medically important event or reaction.

10 The date and/or time of onset is defined as the time post immunization, when dysfunctional labor is diagnosed. This may only be possible to determine in retrospect.

11 Example recovery to pre-immunization health status, spontaneous resolution, therapeutic intervention, persistence of the event, sequelae, death.

12 To determine the appropriate category, the user should first establish, whether a reported event meets the criteria for the lowest applicable level of diagnostic certainty, e.g. Level three. If the lowest applicable level of diagnostic certainty of the definition is met, and there is evidence that the criteria of the next higher level of diagnostic certainty are met, the event should be classified in the next category. This approach should be continued until the highest level of diagnostic certainty for a given event could be determined. If the lowest level of the case definition is not met, it should be ruled out that any of the higher levels of diagnostic certainty are met and the event should be classified in additional categories four or five.

13 If the evidence available for an event is insufficient because information is missing, such an event should be categorized as “Reported dysfunctional labor with insufficient evidence to meet the case definition”.

14 An event does not meet the case definition if investigation reveals a negative finding of a necessary criterion (necessary condition) for diagnosis. Such an event should be rejected and classified as “Not a case of dysfunctional labor”.

15 The end of an episode is defined as the time the event no longer meets the case definition at the lowest level of the definition.

16 The date and/or time of first observation of the first sign or symptom indicative for dysfunctional labor can be used if date/time of onset is not known.

17 The date of diagnosis of an episode is the day post immunization when the event met the case definition at any level.

18 Use of this document should preferably be referenced by referring to the respective link on the Brighton Collaboration website (http://www.brightoncollaboration.org).
