# Supplementary material for: Dysfunctional labor: Case definition & guidelines for data collection, analysis, and presentation of immunization safety data
Source: Vaccine. 2017 Dec 4;35(48Part A):6538–45. doi: 10.1016/j.vaccine.2017.01.050 (PMC5710983; doi:10.1016/j.vaccine.2017.01.050)
Supplement: Supplementary data 1 [file mmc1.docx]

| **Question** | **Review feedback** | | **Authors' comments** |
| --- | --- | --- | --- |
| 1.a. Do you agree with the rationale of the case definition? | Yes | 3 |  |
|  | No | 2 |  |
|  | Do not know | 0 |  |
| 1.b. If no / do not know, explain why. | 1.The authors note that there is no biologic plausibility to a theory of causal relationship of these events to vaccination and no reported [causal][temporal] links either. So the process seems to lack rationale but to surely enhance the risk of reports such common events as dysfunctional labor and vaccination, which are universally temporally related, being reported and confusing the issue. In that light it behooves the authors to include some discussion of how to establish causality or the lack thereof, with appropriate references.  2 .It is noted in para. 2 of preamble that there is a lack of consensus on the criteria. However, the paper includes no mention of how the proposed definitions address that point. In fact, they do not address it, and particularly with regard to limited resource nations, in many of which most pregnancies are unattended by obstetricians or even by trained midwives who can determine centimeters of dilatation over time. In that light, the definitions are not applicable to such areas; some of those in the table, in contrast, are applicable and should be included. |  | 1. Thank you for your comment regarding causal relationship and biologic plausibility. We recognize again the lack of case reports or known linkages between vaccination and dysfunctional labor. In accordance with vaccine safety guidelines, the world health organization recommends the monitoring of a broad array of events that may not have an established or known causal relationship to vaccine use. Though dysfunctional labor is common and therefore likely to be related temporally to vaccine use, that alone is not enough to establish causality. The purpose of data collection for this particular event thus, is likely to help prove a lack of causation rather than the converse. This discussion has been added to the manuscript (Page 2 lines 58-72)  2. Thank you for your comment regarding lack of consensus of criteria and applicability to low resource settings. In formulating these definitions we relied on the expertise of a working group comprised of members from a diverse set of backgrounds including members from low resource settings. The guidelines noted in Table 1 are drawn from available published guidelines from different regions. Criteria used within the case definitions were decided upon using these guidelines as a basis. We acknowledge that the current formulations of these definitions require some form of monitoring during labor thus would not be applicable to woman with unattended deliveries, particularly for the first stage where the rate of cervical dilation is an integral component. The definitions used in the second stage, which are based on time may be applicable for unmonitored deliveries, though require either the woman giving birth or not formally educated birth attendant, if present, to provide some assessment of time. |
| 1.c. Comments? | I would agree that a case definition around labour is required, but "dysfunctional labor" is a rather narrow pathology with a specific focus on a long birth process. Situations such as quick labour (with e.g. ruptures), fluxus (bleeding after delivery) would also be important to assess after recent immunization; basically all events that are abnormal, not only the delayed birth. Understandably, it is difficult to capture all this into one definition, but still I would feel uncomfortable assessing only the prolonged duration of labour. |  | Thank you for comment regarding the scope. We agree that dysfunctional labor is focused on one element of the long birth process. Due to the limited scope of this manuscript we intentionally have maintained this narrow focus. Other manuscripts are in process to address other events or complications that may occur during the birth process. |
|  | None |  |  |
|  | Concern regarding wording in the paragraph explaining the use of regional anesthesia. "Delayed second stage" is a strange wording, I am assuming you are referring to prolonged second stage? and not a delay to onset of second stage? |  | Thank you for the comment. We agree that ‘delayed’ in this context can be confusing and have changed the wording to ‘prolonged’ to clarify that this refers to the duration of the second stage rather than to its onset. See page 4 line 171. |
| 2.a. Does the preamble clearly describe the intended use of the definition? | Yes | 4 |  |
|  | No | 1 |  |
|  | Do not know | 0 |  |
| 2.b. If no / do not know, explain why. | 1. Not stated if search restricted only to English language articles. 2. 1.1 should include medications, recreational drug use, alcohol as possible influences on incidence. 3.The last para. in 1.3 illustrates the conundrum of linking post vaccination event that has no plausible biological link to a particular AE. Is the intended use (or, indeed the inevitable unintended use) going to be the filing of AE reports on every vaccinated pregnant woman who has this AE??? How to avoid that seemingly abortive and counterproductive result? Where are the issues of causality/noncausality proofs discussed? Elsewhere GAIA definitions are supposed to include applicable ones to underdeveloped areas, but here those are eliminated from the definitions as formulated, while some which could be used and are listed in Table 1 are ignored in the final result. |  | 1. Thank you for the comment. The search was not restricted to the English language. This has been clarified in the manuscript (Page 3, line 117-8). 2. We have updated the working in the manuscript to reflect the multiple potential influences on incidence of labor dysfunction (page 3, line 102). 3. Please refer to the comment above on causality and biological link. Please also refer to the comment above on applicability to low resource settings |
| 2.c. Comments? | None |  |  |
| 3.a. Do you agree with Level 1 of diagnostic certainty? | Yes | 4 |  |
|  | No | 1 |  |
|  | Do not know | 0 |  |
| 3.b. If no / do not know, explain why. | 1. Not suitable for underdeveloped nations or areas 2.The AND/OR SECTIONS ARE NOT CLEARLY DELINEATED. For example one could read level 1 as "onset of active stage" AND greater than 2 hrs, etc.  OR  greater than 1 hr of pushing, etc...  OR DOES IT MEAN:  "Onset of active stage AND  either greater than 2 hrs, etc. or greater than 1 hr etc.??? |  | Thank you for this comment. Regarding suitability for underdeveloped nations please refer to our comment above  Regarding the formatting of the definitions we have made changes to the layout and separated out the definition for nulliparous and multiparous women to improve clarity. Please see pages 5-6, lines 246-277 |
| 3.c. Comments? | Although my above comment about the (narrow) scope still stands. |  | Please refer to the comment above on the scope of the manuscript |
|  | I am sure that the authors must have individually managed the occasional patient with intact membranes at almost full cervical dilatation. (lines 10/11), page 5/16. |  | Thank you for this comment. We agree that a woman may progress normally until full dilation with intact membranes and this woman with cervical change at the rate we have defined would not meet the definition for dysfunctional labor. In Level one we included ruptured membranes included to increase the specificity and exclude those women who may have advanced cervical exams without being in labor. In level two this requirement is removed to allow for more sensitivity but less specificity. The wording around excluding the need for ruptured membranes has been clarified and a footnote added. See page 6 Footnote 2 |
|  | Just a spelling error or word missing: "For both levels of diagnostic the woman is in" |  | Changed in manuscript see page 5 line 230 |
| 4.a. Do you agree with Level 2 of diagnostic certainty? | Yes | 3 |  |
|  | No | 1 |  |
|  | Do not know | 1 |  |
| 4.b. If no / do not know, explain why. | See 3b. 1 and 3b2. |  |  |
|  | Please also make sure that the second stage definition for instrumental delivery makes it clear that the included definition is for use of instrumental delivery for labor dystocia and not for all indications which could include fetal status. Some concern regarding the way that you are defining with or without regional analgesia. this differs from the clinical practice standard, so you are embarking a bit into new territory. see comment below. However, it may be particularly useful and simplified given the lack of anesthesia access in many low and mid income countries. |  | Thank you for the comment – this is addressed in the footnote 5 on page 6  Thank you for this comment – we have further explained our rationale on epidural use in the preamble rather than as a footnote. See page 4 line 165-174 |
| 4.c. Comments? | So, you are mandating a higher number of women with regional anesthesia to meet the case definition of dysfunctional labor in the first stage and potentially in the second stage as well. should you also include the caveat about the regional anesthesia prior to the first stage case definition? or how do you plan to deal with those patients in the first stage where regional anesthesia may be the factor modifying the risk of dysfunctional labor? |  | See above |
| 5.a. Do you agree with Level 3 of diagnostic certainty? | Yes | 1 |  |
|  | No | 1 |  |
|  | Do not know | 3 |  |
| 5.b. If no / do not know, explain why. | COULDN'T FIND ANY LEVEL 3 IN DEFINITIONS LISTED OR ON PAGE 9, WHERE LEVELS 1,2,4,AND 5 ARE LISTED WITHOUT 3. |  | Thank you for the comment. The working group consensus is that beyond the criteria specified in level 2, fewer criteria would not provide for a case definition that was specific enough for a study setting thus no level three was defined.  This is discussed on page 4 line 176-8 and 193-195 |
|  | Is there a definition? |  |  |
|  | Not shown, not applicable. |  |  |
|  | no level three in this doc |  |  |
| 5.c. Comments? |  |  |  |
| 6.a. Do you agree with the footnotes of the case definition? | Yes | 3 |  |
|  | No | 2 |  |
|  | Do not know | 0 |  |
| 6.b. If no / do not know, explain why. | CAUSALITY OR LACK THEREOF NEVER MENTIONED AS AN ISSUE TO DEAL WITH |  | Please see notes above regarding causality |
|  | see above re regional anesthesia |  | See comment above regarding the use of regional anesthesia |
| 6.c. Comments? |  |  |  |
| 7.a. Is the definition applicable in your setting? | Yes | 2 |  |
|  | No | 2 |  |
|  | Do not know | 1 |  |
| 7.b. If no / do not know, explain why. | SEE ABOVE COMMENTS |  |  |
|  | I'm an independent medical epidemiologist, not a clinical doctor. |  |  |
|  | we use the definition updated as per Zhang in US (Denver) so people may feel at odds starting with 4cm, but for Guatemala where i also work they would be more comfortable with this case definition as they still use 4cm for active labor. |  | Thank you for this comment. We recognize that using 4cm rather than 6cm as a cut off for established labor might raise some debate. We ultimately decided to use 4cm in our definition as this currently has the broadest applicability internationally and our mandate was to provide a definition without any proscription for intervention. Our interpretation was that the findings by Zhang describe an acceleration at 6cm and this point in cervical dilation was then used as cutoff to prevent unnecessary surgical intervention by ACOG/SMFM but does not necessarily change the definition of established labor. This is clarified in the manuscript on page 4, lines 154-158 |
| 7.c. Comments? | None |  |  |
| 8.a. Do you agree with the guidelines? | Yes | 3 |  |
|  | No | 1 |  |
|  | Do not know | 1 |  |
| ,8.b. If no / do not know, explain why. | 1. On p.7, item 3.1.2, heading should apparently read:  VACCINEE/INFANT, not VACCINE/Control, as data in #s 5,6,7 deal with VACCINEE(mother) and INFANT and controls are never mentioned.  2.Identification by merely initials is totally inadequate due to multiple individuals sharing same initials.  3.Headings should clearly differentiate data needed on mother from data needed on infant. Infant section should include infant name or hospital identifier or code.  4.As always, the vaccine diluent and lot number are omitted.  5.Section 3.1.5. The duration, by definitions has to be up until the end of labor. The rest is boilerplate and should be left out in this duration section. Furthermore, many reporters suggested as sources of the AEFI reports would not have capacity to judge biologic characteristics of anything.  6. 3.2 Data analysis-3.3 data presentation. There is a glaring omission of any discussion of statistics , including inclusion of sample size in reports, statistical power and significance  calculations/methods, etc.  7. Guideline 42: All descriptions of questionnaires, diary cards and report forms should describe how these instruments were validated (in fact they often are used without any validation and are often loaded with leading questions and requests for data that are impossible to collect in a standardized confirmable or reproducible way). This is a major flaw in such studies, usually honored in the breach. This leads to the garbage-in, garbage-out syndrome and is why many vaccine safety epidemiologists sneer at such "data". |  | Thank you for your comment. These guidelines for data collection used in our manuscript follow published guidelines by the Brighton Collaboration for data collection. Please see page 7 lines 291-295  We have made additional changes to respond to the reviewer comments as below:  Page 8 line 325 regarding infant participants  Page 9 line 402-404  Page 10 line 459-460 |
| 8.c. Comments? | See my first comment about the scope. |  |  |
| 9. Any general comments? | 1.Table 1 lists some definitions applicable in resource-limited areas (underdeveloped countries/areas). |  |  |
|  | There is no information about twin pregnancies? Does the time between vaccine and labour also take the pregnancy week into account (37 weeks may go faster than 42 weeks)? Questions to include: what stage of labour, outcome of infant (Apgar, death). There's a mention of 5 categories on page 9, but I only see 4 categories. Grammar needs another check. |  | Thank you for this comment. We restricted these definitions to term singleton pregnancies to avoid potential differences between groups with preterm, postdates or multiple pregnancies. See Page 4 lines 140-147. |
|  | None |  |  |
|  | some spelling errors, spacing non standard |  |  |
|  | in section 3.1.4 consider including date and time of regional anesthesia placement and duration of use and stated indications for procedure. some spelling errors in section 33. Also I hope that when you are collecting medical problems as risk factors for dystocia that you consider collecting Female genital mutilation status and any mental health conditions of the mother as they affect labor progress. |  | Thank you for this comment – we have updated the section on page 9 line 382-383 to include regional anesthesia. Additionally other co-morbidities are collected as part of the standard guidelines on data collection (page 8 lines 328-321). |
| 1.b. If no / do not know, explain why. |  |  |  |
| 1.c. Comments? |  |  |  |
| 2.a. Do you agree with the definition? | Yes | 0 |  |
|  | No | 0 |  |
|  | Do not know | 0 |  |
| 2.b. If no / do not know, explain why. |  |  |  |
| 2.c. Comments? |  |  |  |
| 3.a. Based on your study setting and the procedures / diagnostic requirements for the different levels of certainty in the case definition, would you be able to obtain all levels of certainty? | Yes | 0 |  |
|  | No | 0 |  |
|  | Do not know | 0 |  |
| 3.b. If no / do not know, explain which level(s) would not be applicable and why (e.g. not having all procedures available in countries where study is done / retrospective assessment of charts would not allow information / exclusion / negative criteria to be found consistently) |  |  |  |
| 3.c. Comments? |  |  |  |
| 4.b. If no / do not know, explain why. |  |  |  |
| 4.c. Comments? |  |  |  |
| 5.a. How much would the use of case definitions such as this one potentially improve your study? |  |  |  |
| 5.b. Comments? |  |  |  |
| 6. What additional documents would you wish GAIA to develop for your study in order to achieve harmonisation of safety assessment in maternal immunisation studies? |  |  |  |
| 7. Any other comments you would like to share? |  |  |  |
